# On Diseases of the Bladder

**Published:** 1838-10

**Authors:** 


### PART SECOND.

### Bibliographical Notices.

---

Art. I.—*On Diseases of the Bladder.* By Wm. Coulson, Surgeon. *London,* 1838. 18mo. pp. 153.

It would be well if authors writing on scientific subjects would fulfil one of two conditions, each of which would be admitted by all as a legitimate reason for producing a book: one of these is, to communicate what is new and important; the other, to render more perspicuous or comprehensive a subject, the materials of which are either much scattered or not commonly accessible.

We cannot but entertain considerable doubts whether either of these objects has been attained in the work before us. Not only, as it appears to us, does it contain nothing positively new, but it even involves some things which before were reduced to comparative simplicity. The author's discussions are, moreover, too diffuse; and his distinctions between one disease and another are occasionally so vague and shadowy as to make it difficult to identify them. At best, the volume is but a collection of such observations and cases as have been already recorded, although they may have resulted from the author's own experience; and we have that opinion of Mr. Coulson that we wish he himself had felt that something more would be expected from him than the construction of a pocket compendium of certain of the more prominent and distressing of the diseases to which the urinary bladder is subject.

The work is divided into ten chapters, the subjects of which are—Irritability of the bladder; paralysis; acute inflammation of the mucous tunic; subacute inflammation of the same tunic; acute inflammation of the muscular coat; chronic inflammation of the same; inflammation of the peritoneal coat and of the surrounding cellular tissue; fungus hæmatodes and cancer; foreign bodies in the bladder; operation for stone; wounds and injuries of the bladder.

In the first chapter, after stating the symptoms of irritability of the bladder, and giving cases illustrative of the disease, the author observes that "the distinction between this affection and subacute inflammation of the mucous tunic is, however, easy;" giving the following as his reason for thinking so: "If," he says, "the affection has been of very long standing, the general health (if the disease be irritability) suffers but little; whereas, in chronic inflammation of the bladder, the constitutional powers sooner or later give way." We cannot quarrel with the author for entertaining this opinion; though we should have thought that experience would have taught him, as it has taught us, that it was a quicksand in practice. According to our experience, chronic inflammation of the bladder, accompanied by mucous or muco-purulent discharge although it produces a train of symptoms which are distressing to the patient, does not commonly excite very active sympathetic action in the economy; and the disease remains for many years local, and quite compatible with the continuance of life. We often meet with old men, who, for ten, fifteen, or twenty years, have suffered from chronic inflammation of the bladder, while the general health remains satisfactory: we therefore maintain, that the distinction proposed by the author cannot be safely relied on: indeed, it appears to us that the product of secretion must be regarded at present as the truest test between irritability and inflammation. We would not, however, be understood to deny that cases of chronic inflammation may commence with irritability; that is, there may be, for a certain time, no apparent change of structure: we wish merely to convey an impression that we possess no power of marking out a precise demarcation between the one affection and the other.

Again, we are satisfied that the attempt to make out a distinct series of symptoms, characteristic of inflammation, affecting separately either of the two internal tunics of the bladder, is, as a general rule in practice, utterly vain. How often has the author seen a case of pure and simple inflammation of either the mucous or muscular tunic? We would not deny the occasional existence of such a condition; but we believe it to be so very unfrequent that, if we possessed the power of discrimination, it could be only very rarely exercised, and would therefore have little practical value. We know of two cases in which inflammation affected the peritoneal coat, without attacking either of the others: we know of none in which it has been limited to the muscular tunic; and, although there are cases on record where the mucous tunic was alone affected, yet some of them are unsatisfactory.

Are we to infer, from the author's not formally noticing it, that it is his opinion that chronic inflammation of the mucous membrane of the bladder does not occur? He describes a chronic inflammation of the muscular tunic: if the mucous coat is exempt, upon what circumstance is this caprice of nature founded? We know that mucous tissue, wherever situated, is subject to chronic inflammation, and we know no law specially applicable to the bladder by which it should be excluded. On the contrary, judging from our own experience, we should say that it often occurs in that organ. We believe that our author has confounded it, on the one hand, with irritability, on the other, with subacute inflammation; and we think that several of his own cases may be adduced in support of this view of the subject: and, indeed, his own remarks imply as much; for, in another part of his work, after stating the symptoms of irritability, and giving cases illustrative of the disease, he says, "If the affection has been of very long standing, the general health (if the disease be irritability) suffers but little; whereas, in *chronic* inflammation of the bladder, the constitutional powers sooner or later give way."

Upon the remaining chapters of this work we have no remarks to offer: we have stated their general character in the beginning of this notice. In conclusion, however, we would observe, in justice to the author, that, although this little volume will, in no degree, add to his reputation, it will certainly be useful to the junior members of the profession, who may either not have access to larger treatises, or time or inclination to read them. To this class of readers one portion of Mr. Coulson's labours will be especially useful,—that which indicates the more important of the formulæ commonly employed by the best surgeons of the metropolis in the treatment of these diseases.

